# Punicalin Alleviates OGD/R-Triggered Cell Injury via TGF-*β*-Mediated Oxidative Stress and Cell Cycle in Neuroblastoma Cells SH-SY5Y

**DOI:** 10.1155/2021/6671282

**Published:** 2021-02-12

**Authors:** Tiansong Yang, Qingyong Wang, Yuanyuan Qu, Yan Liu, Chuwen Feng, Yulin Wang, Weibo Sun, Zhongren Sun, Yulan Zhu

**Affiliations:** ^1^First affiliated hospital, Heilongjiang University of Chinese Medicine, Harbin, China; ^2^Heilongjiang University of Chinese Medicine, Harbin, China; ^3^Harbin Medical University, Harbin, China; ^4^Department of Neurology, The Second Affiliated Hospital of Harbin Medical University, Harbin, China

## Abstract

**Purpose:**

The research aimed to identify the active component from *Punica granatum* L. to alleviate ischemia/reperfusion injury and clarify the underlying mechanism of the active component alleviating ischemia/reperfusion injury.

**Materials and Methods:**

The SH-SY5Y cell model of oxygen-glucose deprivation/reoxygenation (OGD/R) was established to simulate the ischemia/reperfusion injury. According to the strategy of bioassay-guided isolation, the active component of punicalin from *Punica granatum* L. was identified. Flow cytometry and Western blotting were employed to evaluate the effects of OGD/R and/or punicalin on cell cycle arrest. Immunofluorescence assay was applied to assess the nucleus translocation. The relative content of ROS and GSH and the enzyme activities of CAT and SOD were examined using ELISA.

**Results:**

The data of bioassay-guided isolation showed that punicalin from *Punica granatum* L. could alleviate OGD/R-induced cell injury in SH-SY5Y cells. Flow cytometry analysis and Western blotting for probing the expression of CDK1, p-CDK1, cyclin B1, and p21 revealed that punicalin could relieve OGD/R-induced cell cycle G0/G1 arrest. Additionally, immunofluorescence assay and Western blotting for probing the expression of TGF-*β* and p-Smad2/p-Smad3 showed that punicalin could relieve the OGD/R-induced TGF-*β*/Smad pathway. Furthermore, the TGF-*β*/Smad pathway inhibitor of LY2157299 was employed to confirm that the TGF-*β*/Smad pathway is crucial to the effect of punicalin. At last, it was indicated that punicalin could relieve OGD/R-induced oxidative stress.

**Conclusion:**

Punicalin, an active component from *Punica granatum* L., was identified as a protective agent to alleviate the OGD/R-induced cell injury, which could exert the protective effect via TGF-*β*/Smad pathway-regulated oxidative stress and cell cycle arrest in SH-SY5Y cells.

## 1. Introduction

Cerebral ischemic stroke is considered as a brain injury disease with the symptoms of hemiplegia, cognitive impairment, and disability, which are caused by clogging blood vessel leading to insufficiency of cerebral blood supply [1]. It accounted for approximately three million individual deaths all over the world in 2015, and the mortality is still rising year by year, seriously threatening human life and safety. Thrombolysis is one of the main strategies to treat ischemic stroke, and recombinant tissue plasminogen activator (rtPA) as a thrombolysis agent is now usually employed to alleviate the ischemic stroke through dissolving intravascular thrombus [2, 3]. Although the use of rtPA provides a remission in ischemic symptoms, it also triggers more severe side effects of brain damage and neuronal disorder referred to as ischemia/reperfusion injury (IRI). Several shreds of evidence indicated that oxidative stress, apoptosis, cell cycle arrest, and calcium overload could be involved in IRI [4, 5]. However, the complex pathological mechanisms remain elusive, which also restricted the development of IRI medicinal agent. Hence, it is urgent and significant to elucidate the IRI progress and identify the potential candidate for clinical treatment in IRI.

The TGF-*β*/Smad pathway is recognized as a process of signaling cascade response to the growth factor for maintaining the individual's dynamic balance of physiology and pathology. It plays many crucial roles in cell proliferation, apoptosis, oxidative stress, and inflammation, participating in a series of disease events such as cancer, diabetes, and cardiocerebrovascular disease [6, 7]. Recent shreds of evidence showed that TGF-*β* could serve as an injury-related cytokine involving brain injury and neurodegenerative diseases. It is reported by Abdel et al. that propolis could relieve cerebral injury via the regulation of the TGF-*β*/Smad pathway [8]. Another study showed that the TGF-*β*/Smad pathway involved in the cerebral IRI after isoflurane exposure [9]. These findings imply us that the TGF-*β*/Smad pathway could be one of the crucial parts in IRI progress. Therefore, identifying the TGF-*β* inhibitor would be a potential strategy to develop a medicinal agent for IRI treatment.

*Punica granatum* L. (PGL), also named pomegranate, has been wildly recognized as an edible fruit for hundreds of years in the earth. Recently, increasing shreds of evidence revealed that PGL has many bioactivities including anti-inflammation and antioxidation and exert a vast benefit in treatment and prevention of diseases such as diabetes, cancer, cardiovascular disease, and ischemia injury [10, 11]. The component analysis indicated that there are a series of active compounds containing procyanidin, corilagin, ellagic acid, and punicalin exiting in PGL. Among these, procyanidin reported to have a protective effect against brain damage in mice via alleviating cell apoptosis, oxidative stress, and induced angiogenesis [12, 13]. Corilagin possessed oxidation-inhibiting and angiogenic-evoking effects to ameliorate cerebral ischemia [14], and ellagic acid could relieve cerebral injury through mediating neuron apoptosis and restoring brain-blood barrier (BBB) [15]. The information as mentioned earlier demonstrated that PGL could be a valuable treasure chest of potential medicinal agents' development for the treatment of diseases especially IRI.

Here, we established an oxygen-glucose deprivation/reoxygenation (OGD/R) model in human neuroblastoma cell SH-SY5Y to simulate IRI, identify a TGF-*β* inhibitor of punicalin alleviating OGD/R-induced neuroblastoma injury from PGL extraction, and elucidate the mechanism of punicalin-suppressing cell injury via TGF-*β*-mediated oxidative stress and cell cycle arrest. The present study revealed the role of TGF-*β* in IRI development and treatment and provided a novel therapeutic approach for IRI.

## 2. Materials and Methods

### 2.1. Chemicals and Reagents

DMEM medium (Cat No. C11995) and FBS (Cat No.10099-141) were purchased from Gibco (USA). MTT reagent and propidium iodide (PI, Cat No. P8080) were obtained from Solarbio (China). The primary antibody of CDK1 (Cat No. ab133327), p-CDK1 (Cat No. ab201008), cyclin B1 (Cat No. ab32053), p21 (Cat No. ab109520), TGF-*β* (Cat No. ab215715), and Smad 2/Smad3 (Cat No. ab202445) and the secondary antibody were purchased from Abcam (USA). The dyes of DAPI (Cat No. 268298) were purchased from Sigma (USA). The ELISA assay kits of ROS (Cat No. E004-1-1), CAT (Cat No. A007-1-1), GSH (Cat No. A005-1-2), and SOD (Cat No. A001-3-2) were obtained from Nanjing Jiancheng Bioengineering Institute (China). The LY2157299 (Cat No. HY-13226), a TGF-*β* inhibitor, was purchased from MedChemExpress (MCE). The organic solvents ethanol, petroleum ether, chloroform, and ethyl acetate were purchased from Sinopharm Chemical Reagent Co., Ltd (China).

### 2.2. Preparation of *Punica granatum* L. Extraction

The fruit of *Punica granatum* L. was purchased from a farmers' market in Harbin city, and the seeds were taken out. The collected seeds were dried in a vacuum drying oven and ground to powder using crushing machinery. The powder (500 g) was refluxed and extracted with 2.5 L 60% ethanol for 2 hours, and the extraction was repeated thrice. The extraction solution was merged and evaporated in rotary evaporation to no alcohol taste. After that, the ethanol extraction was furthermore extracted with petroleum ether, chloroform, and ethyl acetate, respectively, to obtain three fractions of PE, CF, and EA. The three fractions were dried to solid in rotary evaporation, and the active compound was identified according to bioactivity-guided isolation.

### 2.3. Cell Culture

Human neuroblastoma SH-SY5Y cells were purchased from Cell Resource Center, Shanghai Institutes for Biological Sciences, and Chinese Academy of Sciences. The SH-SY5Y cells were cultured in DMEM mediums with 10% FBS and the condition of 5% CO_2_ at 37°C.

### 2.4. Construction of the OGD/R-Induced Cellular Injury Model

The SH-SY5Y cells were seeded into a 6-well plate and cultured in the DMEM medium with glucose-free. The culture conditions are 5% CO_2_, 1% O_2_, and 94% N_2_ at 37°C for 6 hours. And then, the medium was replaced with normal DMEM medium and cultured in the normal atmosphere of 5% CO_2_ for another 24 hours to reoxygenate.

### 2.5. MTT  Assay

SH-SY5Y cells were seeded into the 96-well plate and cultured for 12 hours. And then, the seeded cells were treated with OGD/R and *Punica granatum* L./punicalin at the response concentration for the specific time. After treating, the cells were added with 20 *μ*L MTT (5 mg/mL), cultured for another 3 hours, and diluted with 100 *μ*L DMSO, and the OD value was read using a microplate reader (Peiqing, China). The proliferation rate was calculated using Microsoft Excel software as

(1) $$\begin{array}{c} \text{Proliferation rate}=\frac{\left({\text{OD}}_{\text{sample}}-{\text{OD}}_{\text{blank}}\right)}{\left({\text{OD}}_{\text{control}}-{\text{OD}}_{\text{blank}}\right)}\times 100\%. \end{array}$$

### 2.6. EdU Incorporation Assay

SH-SY5Y cells were seeded into a 12-well plate and cultured for 12 hours. And then, the seeded cells were treated with OGD/R and *Punica granatum* L./punicalin at the response concentration for the specific time. After treating, the cells were added with 300 *μ*L EdU medium (50 *μ*M), cultured for another 2 hours, and washed with PBS twice. The cell images were captured with a fluorescence microscope (Nikon, Japan).

### 2.7. Cell Cycle Distribution Analysis

SH-SY5Y cells were seeded into a 6-well plate and cultured for 12 hours. And then, the seeded cells were treated with OGD/R and punicalin for the specific time. After treating, the cells were digested into the state of signal cells, collected the cells into 1.5 mL tubes, fixed with precold 70% alcohol at room temperature for 30 min, and stained with PI for another 30 min. The stained cells were detected by flow cytometry (Beckman, USA).

### 2.8. Western Blotting

SH-SY5Y cells were seeded into a 6-well plate and cultured for 12 hours. And then, the seeded cells were treated with OGD/R and punicalin for the specific time. After treating, the cells were lysed using RIPA lysis buffer, centrifuged, and harvested the total protein. Total protein was subjected to the SDS-PAGE for separation, transferred protein to PVDF membrane, blocked PVDF membrane with 5% fat-free milk, incubated with primary antibody of CDK1, p-CDK1, cyclin B1, p21, TGF-*β*, and p-Smad2/Smad3 overnight at 4°C, washed with TBST for three times, incubated the response secondary antibody at room temperature for 1 hour, washed for three times, and at last probed with ECL reagents.

### 2.9. Immunofluorescence

SH-SY5Y cells were seeded to the slices in a 12-well plate and cultured for 12 hours. And then, the seeded cells were treated with OGD/R and punicalin for the specific time. After treating, the slices were fixed with formaldehyde, perforated with triton X-100 solution, fixed with BSA, incubated with primary antibody of Smad3 and secondary antibody at room temperature, and stained with DAPI and sealed. At last, the slices were examined using a confocal microscope.

### 2.10. Statistical Analysis

The data are expressed as the mean ± SD, and the analysis is performed with SPSS software (USA). Statistical significance is defined as *p* < 0.05.

## 3. Results

### 3.1. The Effects of *Punica granatum* L. Extraction on OGD/R-Induced Cellular Injury in SH-SY5Y Cells

**Figure 1:**
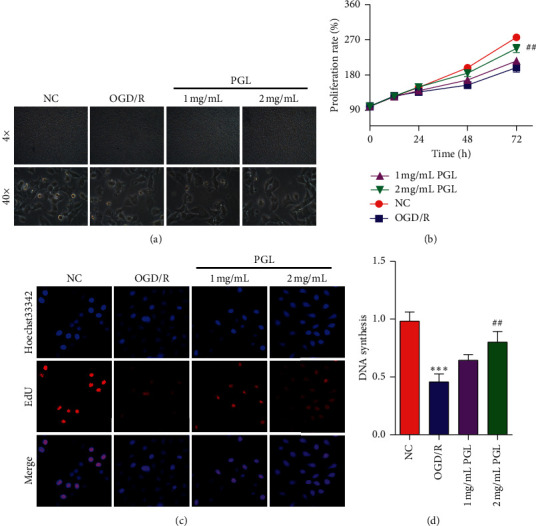
The extraction of *Punica granatum* L. alleviates OGD/R-induced cellular injury in SH-SY5Y cells. (a) The representative image of SH-SY5Y cells subjecting to OGD/R and/or *Punica granatum* L. extraction. (b) The proliferation rate of cells. (c) DNA synthesis of cells. PGL, *Punica granatum* L. ^*∗∗∗*^*p* < 0.001 compared to the normal control, and ^##^*p* < 0.01 compared to the OGD/R group.

The change of cellular morphology and proliferation rate is the appearance indicators to assess cell damage. Microscope photograph (Figure 1(a)) showed that the outline of SH-SY5Y cells subjecting to OGD/R treatment was dispersive and ambiguous, and the amount was declined compared with that of SH-SY5Y cells in the normal control group (NC). *Punica granatum* L. extraction at different concentrations of 1 mg/mL and 2 mg/mL ameliorated the cellular morphology change induced by OGD/R. The MTT assay (Figure 1(b)) revealed that the living cell amount subjecting to the supplement of 2 mg/mL *Punica granatum* L. extraction for 24, 48, and 72 hours was close to the amount in normal control groups and markedly more than that of OGD/R groups (^#^*p* < 0.05 for 48 hours and ^##^*p* < 0.01 for 72 hours). DNA synthesis determines the cellular proliferation and amounts, indirectly reflecting the cell damage. Furthermore, the EdU incorporation assay was employed to evaluate DNA synthesis. As shown in Figure 1(c), the proportion of EdU incorporation in the OGD/R group was significantly suppressed compared to that in the normal control group (^*∗∗∗*^*p* < 0.001) in SH-SY5Y cells. *Punica granatum* L. extraction administration at different doses ameliorated the suppression induced by OGD/R, and 2 mg/mL *Punica granatum* L. extraction raised the EdU incorporation proportion from 0.46 to 0.80 (^##^*p* < 0.01). The results demonstrated that *Punica granatum* L. extraction could alleviate OGD/R-induced cellular injury in SH-SY5Y cells.

### 3.2. Identification of Punicalin as an Active Component from *Punica granatum* L. Extraction

**Figure 2:**
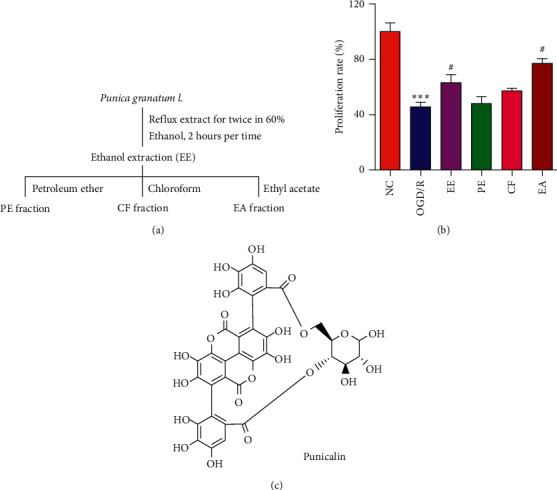
Identifying punicalin as an active component from *Punica granatum* L. (a) The separation process of *Punica granatum* L. (b) The proliferation rate of cells subjecting to the fractions of *Punica granatum* L. (c) The structure of punicalin. ^*∗∗∗*^*p* < 0.001 compared to the normal control, and ^#^*p* < 0.05 compared to the OGD/R group.

The bioassay-guided isolation was employed to determine the active component with the potential of protection against OGD/R-induced cellular injury from *Punica granatum* L. The powdered sample was extracted with ethanol to obtain ethanol extract (EE). The resulted ethanol extract was fractionated petroleum ether (PE), chloroform (CF), and ethyl acetate (EA). The extraction route is shown in Figure 2(a). Then, the protective effect of the ethanol extraction and the three fractions against cell injury were evaluated by the proliferation test. As shown in MTT data of Figure 2(b), OGD/R suppressed the proliferation of human neuroblastoma SH-SY5Y cells (^*∗∗∗*^*p* < 0.001), and the ethanol extraction and EA fraction exhibited a particular alleviation of OGD/R-induced proliferation suppression (^#^*p* < 0.05). Next, the EA fraction was subjected to column chromatography on silica gel with the mobile phase of the chloroform-methanol solution and preparative high-performance liquid phase (pHPLC) with the mobile phase of methanol-water to obtain several compounds. The protection activities of the isolated compounds against cell injury were detected by the MTT assay (the data were not shown). The expected active compound was identified as punicalin, and the structure is shown in Figure 2(c).

### 3.3. Effect of Punicalin on OGD/R-Induced Cell Cycle Arrest

**Figure 3:**
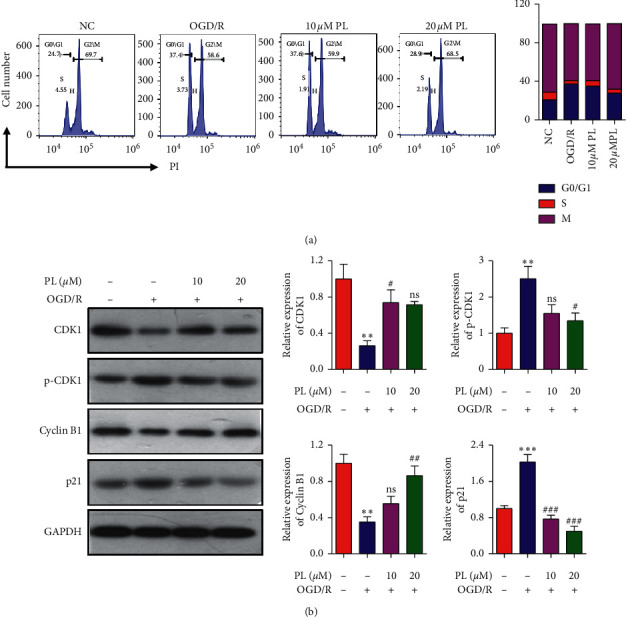
Punicalin alleviates OGD/R-induced cell cycle arrest in SH-SY5Y cells. (a) The cell cycle distribution of cells subjecting to OGD/R and/or punicalin. (b) The expression of cell cycle G1 phase-related proteins of CDK1, p-CDK1, cyclin B1, and p21. PL, punicalin. ^*∗∗*^*p* < 0.01 compared to the normal control, ^#^*p* < 0.05, ^##^*p* < 0.01, and ^###^*p* < 0.001 compared to the OGD/R group, and ns represents not significant.

In order to better understand the role of punicalin in human neuroblastoma SH-SY5Y cells subjecting to OGD/R, cell cycle distribution and its marker proteins were tested, respectively, by flow cytometry and Western blotting. Flow cytometry (Figure 3(a)) revealed that the cells treated by OGD/R exhibited G0/G1 arrest and the ratio of G0/G1 phase rising from 24.7% to 37.4% compared to the normal control group. After the preexposure of punicalin at different concentrations, the ratio of the G0/G1 phase of 10 *μ*M and 20 *μ*M was, respectively, 37.6% and 28.9%, and a high dose of punicalin alleviated the rising of G0/G1 phase ratio in the OGD/R group. The protein marker expression reflecting G0/G1 phase progress (Figure 3(b)) indicated that OGD/R could suppress the expression of cyclin B1 and cyclin-dependent kinases CDK1 and induce the expression of p21 and phosphorylation of CDK1. Punicalin at 20 *μ*M ameliorated OGD/R-mediated expression of cyclin B1, CDK1, p21 and phosphorylation of CDK1, which is consistent with the data of cell cycle arrest. These results demonstrated that punicalin could alleviate OGD/R-induced cell cycle G0/G1 arrest in SH-SY5Y cells.

### 3.4. Effect of Punicalin on the TGF-*β* Signal Pathway in SH-SY5Y Cells

**Figure 4:**
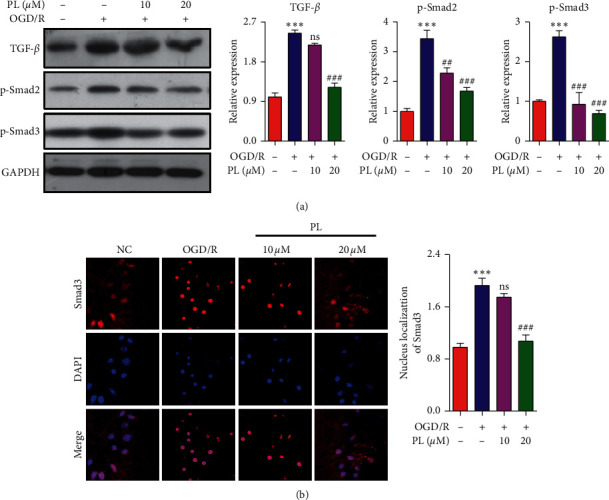
Punicalin mediates the TGF-*β*/Smad pathway in SH-SY5Y cells. (a) The expression of TGF-*β*/Smad pathway proteins of TGF-*β* and p-Smad2/p-Smad3. (b) Immunofluorescence images of Smad3 for nucleus translocation. PL, punicalin. ^*∗∗∗*^*p* < 0.001 compared to the normal control, ^##^*p* < 0.01 and ^###^*p* < 0.001 compared to the OGD/R group, and ns represents not significant.

The TGF-*β* signal pathway is usually activated by biological stimuli, causing the phosphorylation and nucleus localization of Smad2 and Smad3, following inducing related genes expression, and subsequently leading to the occurrence of physiopathology contain IRI. To evaluate the effect of the identified candidate punicalin on the TGF-*β* signal pathway, Western blotting and the immunofluorescence assay were used to examine the level of TGF-*β*, phosphorylation of Smad2 and Smad3, and nucleus localization of Smad3.  Figure 4(a) indicates that OGD/R induced the TGF-*β* expression compared to the normal control group and 20 *μ*M of punicalin inhibited the induction of TGF-*β* expression by OGD/R in SH-SY5Y cells. Similarly, the phosphorylation of Smad2 and Smad3 were induced by OGD/R, and a high dose of punicalin ameliorated the induction of phosphorylation. Furthermore, immunofluorescence image (Figure 4(b)) showed that OGD/R promoted the translocation of Smad3 from the cytoplasm to nucleus; meanwhile, punicalin at 20 *μ*M concentration alleviated the nucleus translocation of Smad3 promoted by OGD/R. Taken together, OGD/R could activate the TGF-*β* signal pathway, and punicalin possesses the ability to attenuate the activation of the TGF-*β* pathway by OGD/R in SH-SY5Y cells.

### 3.5. The  Role of the TGF-*β* Pathway in the Effect of Punicalin on OGD/R-Induced Cellular Injury

**Figure 5:**
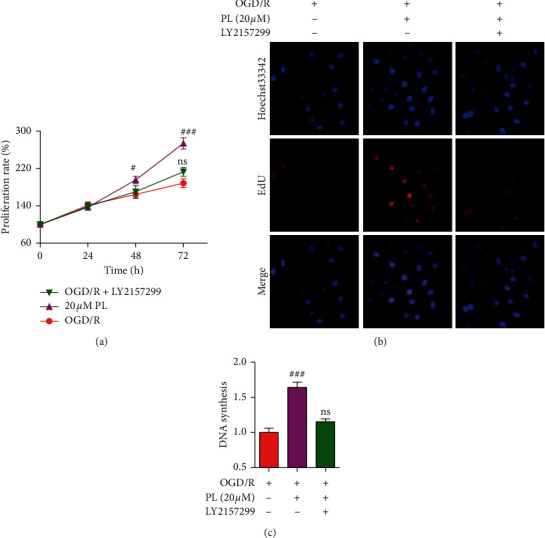
TGF-*β*/Smad pathway is crucial to the effect of punicalin on OGD/R-induced neuroblastoma injury. (a) The cell proliferation rate of cell subjecting OGD/R, punicalin, and LY2157299. (b) The images of EdU staining for DNA synthesis rate in SH-SY5Y cells (c) The statistics of DNA synthesis rate. LY2157299, an inhibitor of TGF-*β*/Smad pathway. PL, punicalin. ^#^*p* < 0.05 and ^###^*p* < 0.001 compared to the OGD/R group, and ns represents not significant.

The abovementioned data demonstrated that punicalin could relieve OGD/R-induced neuroblastoma injury and the TGF-*β* signal pathway. Combined with the shreds of evidence of TGF-*β*-mediating brain injury progress, it has speculated that the TGF-*β* pathway maybe involved in the effect of punicalin on OGD/R-induced neuroblastoma injury. To confirm the role of the TGF-*β* pathway in punicalin alleviating OGD/R-induced cellular injury, LY2157299 was employed as a tool agent inhibiting the TGF-*β* pathway in SH-SY5Y cells. As shown in line chart by the MTT assay (Figure 5(a)), the proliferation ratio in the punicalin group was higher than that in the OGD/R group, and the *p* value was, respectively, ^#^*p* < 0.05 for 48 hours and ^###^*p* < 0.001 for 72 hours. Combined supplement of punicalin and LY2157299 exhibited only a litter promotion to cellular proliferation compared to the OGD/R group and no significant difference even for 72 hours in SH-SY5Y cells. Furthermore, the EdU incorporation assay (Figure 5(b)) showed that punicalin significantly raised the DNA synthesis compared to the OGD/R group (^###^*p* < 0.001), and the combined supplement of punicalin and LY2157299 had no raised effect on DNA synthesis. These results demonstrated that the TGF-*β* pathway is involved in the effect of punicalin alleviating OGD/R-induced neuroblastoma injury in SH-SY5Y cells.

### 3.6. Effect of Punicalin on OGD/R-Induced Oxidative Stress

**Figure 6:**
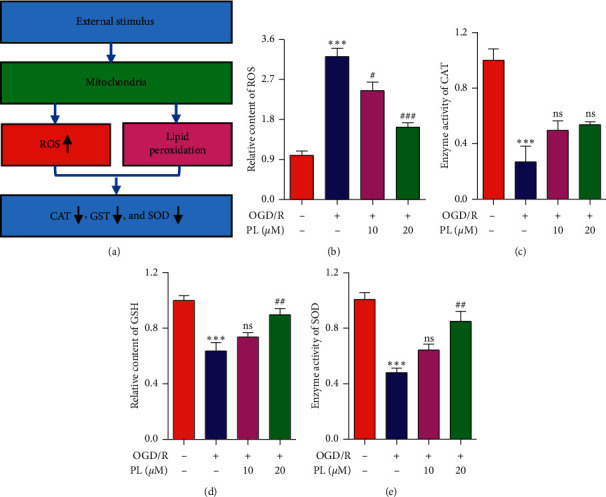
Punicalin alleviates OGD/R-induced oxidative stress in SH-SY5Y cells. (a) The mechanism of oxidative stress. (b) The relative content of ROS. (c) The enzyme activities of CAT. (d) The relative content of GSH. (e) The enzyme activity of SOD. PL, punicalin. ^*∗∗∗*^*p* < 0.001 compared to the normal control, ^#^*p* < 0.05, ^##^*p* < 0.01, and ^###^*p* < 0.001 compared to the OGD/R group, and ns represents not significant.

**Figure 7:**
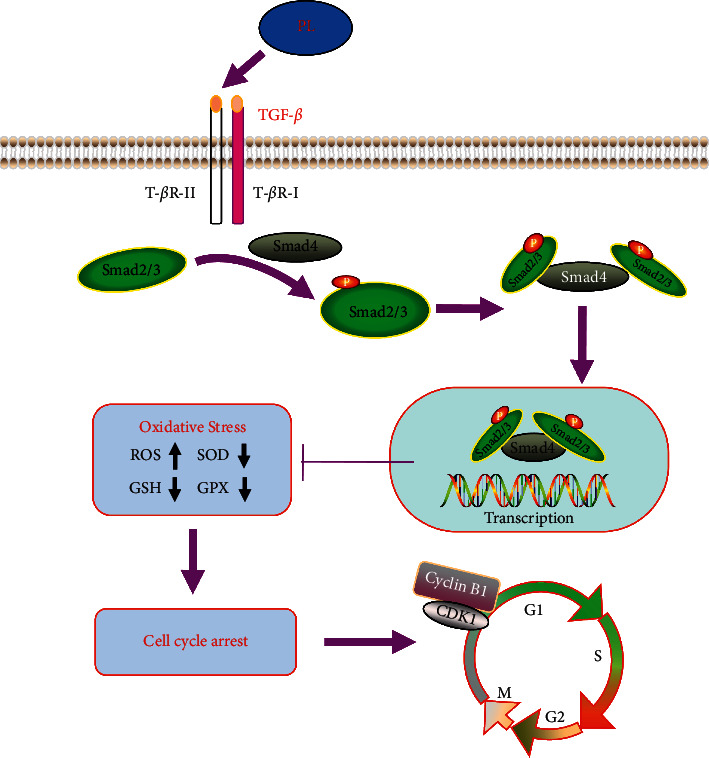
The mechanism of punicalin attenuating OGD/R-induced neuroblastoma injury. PL, punicalin.

To furthermore study the effect of punicalin on oxidative stress during OGD/R-induced neuroblastoma injury, the indicators of ROS, CAT, GST, and SOD reflecting the oxidative stress level (Figure 6(a)) were detected using the ELISA assay. As seen in Figures 6(b)–6(e), OGD/R raised the relative content of ROS, suppressed the relative level of CAT and GSH, and reduced the enzyme activity of SOD compared to the normal control group. Expectedly, punicalin inhibited the induction of ROS content by OGD/R and alleviated the inhibition of CAT and GSH level and SOD enzyme activity. These data implied that punicalin could ameliorate OGD/R-induced oxidative stress in human neuroblastoma SH-SY5Y cells The scheme summarizing the mechanism of punicalin attenuating OGD/R-induced neuroblastoma injury is shown in Figure 7.

## 4. Discussion

Ischemia/reperfusion injury is considered as a brain and blood vessel complication trigged by the thrombolysis treatment to ischemic stroke, which has given a severe threat to human health and happiness. Recent research studies have shown that several traditional Chinese medicines and edible plants, such as Qingkailing injection [16], Yiqi Tongluo granule [17], and *Crepidiastrum denticulatum* extract [18], could relieve ischemia/reperfusion injury. *Punica granatum* L. is an edible fruit with plenty of bioactivities for hundreds of years. It has been reported that *Punica granatum* L. could protect against the injury of the lung, kidney, and myocardium [10, 19]. However, the effect of *Punica granatum* L. extraction and its active components on ischemia/reperfusion injury remains rarely reported. In the current research, the OGD/R model in SH-SY5Y cells was established to simulate IRI. It was applied to screen the protective agents of IRI from *Punica granatum* L. with the strategy of bioassay-guided isolation. Interestingly, an active component of punicalin was found to inhibit OGD/R-induced cellular injury. The finding expands the nutritive value of *Punica granatum* L. and also provides a possible and alternative chance to the prevention and treatment to ischemia/reperfusion injury.

Punicalin is recognized as one member of tannins family, which exists explicitly in the husks, seeds, and leaves of *Punica granatum* L. The pharmacological studies show that punicalin possessed the function of resisting oxidation, protecting against liver damage and removing inflammation [20, 21]. It has been reported that punicalin could alleviate the acetaminophen or carbon tetrachloride-induced liver damage via its antioxidative and hepatoprotective activities [22, 23]. However, the effect of punicalin on ischemia/reperfusion injury and its underlying mechanism is poorly understood. In this research, we found that punicalin might alleviate the OGD/R-induced cellular injury via TGF-*β* pathway-mediated oxidative stress and cell cycle in SH-SY5Y cells.

The TGF-*β*/Smad pathway widely exists in the variety of tissues and cells, which plays a crucial role in the progression of cancer, diabetes, and cerebral injury. The TGF-*β*/Smad pathway induces the phosphorylation of Smad2/smad3 and triggers the shift of Smad3 from the cytoplasm to the nucleus, following evokes related gene expression, and contributes to the pathological process. In the present research, we found OGD/R induced the expression of TGF-*β*, the phosphorylation of Smad2/Smad3, and nucleus translocation in SH-SY5Y cells; meanwhile, punicalin reversed these changing trends. The results demonstrated that the TGF-*β*/Smad pathway might take part in the OGD/R-induced cellular injury and punicalin could alleviate the cellular injury via the TGF-*β*/Smad pathway.

The cell cycle is characterized by a biological process dividing a cell into two daughter cells, which involves the plentiful of regulatory protein and programmed events including the expression and interaction of CDKs and cyclin [24]. It participates in all of the physiology behaviors of differentiation, proliferation, development, and growth from the fertilized eggs and embryo to the individual death [24–26]. The dysregulation of cell cycle would lead to many diseases of cancer and ischemia/reperfusion injury [27–29]. Several shreds of evidence have also shown that the TGF-*β*/Smad pathway could exert the regulatory effect of cell cycle to control cell proliferation and cell death [30, 31]. In the current research, cell cycle distribution analysis displayed that OGD/R induced the cell cycle G1 phase arrest and punicalin could alleviate the induced cell cycle arrest. It was further confirmed that punicalin could reverse OGD/R-regulated expression of CDK1 and cyclin B1, the cell cycle G1 phase-related protein. Combined with the above data of the TGF-*β*/Smad pathway, it is speculated that the TGF-*β*/Smad pathway might involve in the OGD/R-induced cell cycle G1 arrest, and punicalin could alleviate OGD/R-evoked cellular injury via the TGF-*β*/Smad pathway-mediated cell cycle arrest.

Oxidative stress is considered as one of the critical contributions in individual aging and disease progression. When the balance of oxidization in the body is tipped, it would trigger numerous of infiltrating inflammation of neutrophil, produce many oxygen free radicals, and cause the dysfunction of macromolecules including protein, DNA, and lipid, named oxidative stress. Increased ROS content, decreased GSH content, and SOD enzyme activity are the indicators of oxidative stress. Emerging shreds of evidence showed that ischemia/reperfusion injury is associated with oxidative stress. Many agents, including dioscin, *α*-tocopherol, and irisin, were reported to exert protective effects against ischemia/reperfusion injury via oxidative stress [32–34].

Additionally, there is an acceptable crosstalk between oxidative stress and the TGF-*β*/Smad pathway. It had been reported that the TGF-*β*/Smad pathway could regulate many pathological behaviors related to oxidative stress injury. Our data displayed that OGD/R raised the ROS content and inhibited the GSH content and the enzyme activities of CAT and SOD, while punicalin relieved the regulation of OGD/R in SH-SY5Y cells. Together, these results revealed that punicalin might alleviate OGD/R-evoked cellular injury via TGF-*β*/Smad pathway-mediated oxidative stress.

## 5. Conclusions

In conclusion, the present research identified punicalin as the active component from *Punica granatum* L. to protect against OGD/R-induced cell injury and clarified the underlying mechanism that punicalin could alleviate OGD/R-induced cell injury via TGF-*β*/Smad pathway-regulatory oxidative stress and cell cycle arrest. The research expands the nutritive value of *Punica granatum* L., improves the understanding to the bioactivities and mechanism of punicalin, and provides a potential and alternative choice to the prevention and treatment to ischemia/reperfusion injury.

## Data Availability

The data used to support the findings of this study are included within the article.
