# Erratum to “Punicalin Alleviates OGD/R-Triggered Cell Injury via TGF-*β*-Mediated Oxidative Stress and Cell Cycle in Neuroblastoma Cells SH-SY5Y

**DOI:** 10.1155/2021/9840240

**Published:** 2021-04-19

**Authors:** Tiansong Yang, Qingyong Wang, Yuanyuan Qu, Yan Liu, Chuwen Feng, Yulin Wang, Weibo Sun, Zhongren Sun, Yulan Zhu

**Affiliations:** ^1^First Affiliated Hospital, Heilongjiang University of Chinese Medicine, Harbin, China; ^2^Heilongjiang University of Chinese Medicine, Harbin, China; ^3^Harbin Medical University, Harbin, China; ^4^Department of Neurology, The Second Affiliated Hospital of Harbin Medical University, Harbin, China

In the article titled “Punicalin Alleviates OGD/R-Triggered Cell Injury via TGF-*β*-Mediated Oxidative Stress and Cell Cycle in Neuroblastoma Cells SH-SY5Y” [1], the Author's Contributions section is missing. The corrected section appears as follows:

## References

[1] Yang T., Wang Q., Qu Y. (2021). Punicalin Alleviates OGD/R-Triggered Cell Injury via TGF-*β*-Mediated Oxidative Stress and Cell Cycle in Neuroblastoma Cells SH-SY5Y. *Evidence-Based Complementary and Alternative Medicine*.

